# First Report of Hematosalpinx in a Cat With a Large Hematoma and Severe Anemia

**DOI:** 10.1155/crve/7187009

**Published:** 2025-02-13

**Authors:** Teruo Itoh, Atsuko Kojimoto, James Chambers, Kazuyuki Uchida, Hiroki Shii

**Affiliations:** ^1^Aoba Animal Hospital, Miyazaki, Japan; ^2^Division of Animal Medical Research, Hassen-kai, Miyazaki, Japan; ^3^Department of Veterinary Pathology, Graduate School of Agricultural and Life Sciences, The University of Tokyo, Tokyo, Japan

## Abstract

A 6-year-old, intact female cat presented with sudden collapse. Clinical examinations indicated severe regenerative anemia and a large abdominal mass containing fluid. An exploratory laparotomy revealed a large cystic lesion at the cranial end of the right uterine horn. Ovariohysterectomy was performed, and the cat made a full recovery. The cystic lesion contained a blood clot and 102 mL of blood. Histological examination confirmed that the hemorrhagic lesion was located within the fallopian tube. The thickened uterus with severe vasodilation was histologically diagnosed as endometrial hyperplasia with congestion. This is the first reported case of hematosalpinx in a cat, a rare condition previously described in humans.

## 1. Introduction

Hematomas occurring in the ovaries, uterus, or their surrounding areas are rare conditions. In the veterinary field, ovarian hematomas have been reported in horses [1] and cows [2], uterine hematomas in dogs [3–6] and rabbits [7], and broad ligament hematomas in horses [8] and cows [9]. Additionally, hematosalpinx (fallopian tube hematoma) has been documented in humans [10–15], but there are no such reports in veterinary cases. Here, we report a case of hematosalpinx in a cat, characterized by a large hematoma in the fallopian tube and severe anemia.

## 2. Case Presentation

**Figure 1:**
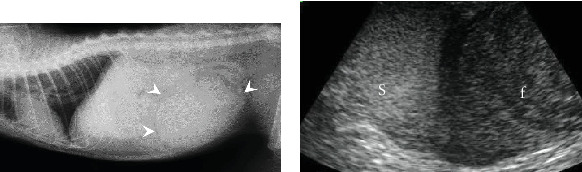
(a) Lateral radiographic image of the cat showing a large round lesion in the caudal abdomen (arrowheads). (b) Ultrasonographic image of the lesion, revealing a hypoechoic fluid component (f) and a relatively hyperechoic solid lesion (s).

A 6-year-old, intact female Japanese domestic cat weighing 4.1 kg presented with sudden collapse. The cat's reproductive and breeding history is unknown, but it had been strictly indoors for the past 11 months, with no chance of mating or pregnancy. On physical examination, the cat had a heart rate of 150 bpm, a rectal temperature of 33.2°C, pale mucous membranes, and weak femoral pulses. Radiographs showed a 10-cm round lesion in the caudal abdomen (Figure 1(a)). Ultrasonography of the same region revealed both a hypoechoic fluid component and a relatively hyperechoic solid lesion (Figure 1(b)). Blood tests indicated severe anemia (hematocrit 15%) with a regenerative response on the blood smear, thrombocytopenia (4.9 cells/*μ*L), and moderate azotemia (BUN 72 mg/dL, creatinine 2.7 mg/dL). Tests for feline immunodeficiency virus antibodies and feline leukemia virus antigen were negative.

Suspecting a neoplastic mass with acute uterine hemorrhage, we administered rapid fluid infusion for 20 min, followed by emergency laparotomy. A large cystic lesion was identified at the cranial end of the right uterine horn, and an ovariohysterectomy was performed. The postoperative course was favorable, with the hematocrit value (28%) and platelet count (26.6 cells/*μ*L) increasing by the 7th day. Blood sex hormone concentrations were not measured. Blood coagulation tests conducted on the 14th day were all within normal ranges, and no abnormalities related to bleeding have been observed in the 4 years after surgery.

**Figure 2:**
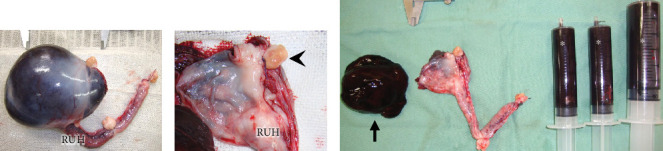
Gross images of the excised ovaries and uterus. (a) A large cystic lesion was formed at the end of the right uterine horn (RUH). (b) The emptied cystic lesion is located between the right uterine horn (RUH) and the grossly normal right ovary (arrowhead). (c) The cystic lesion contains a large blood clot (arrow) and 108 mL of noncoagulated blood (asterisks).

**Figure 3:**
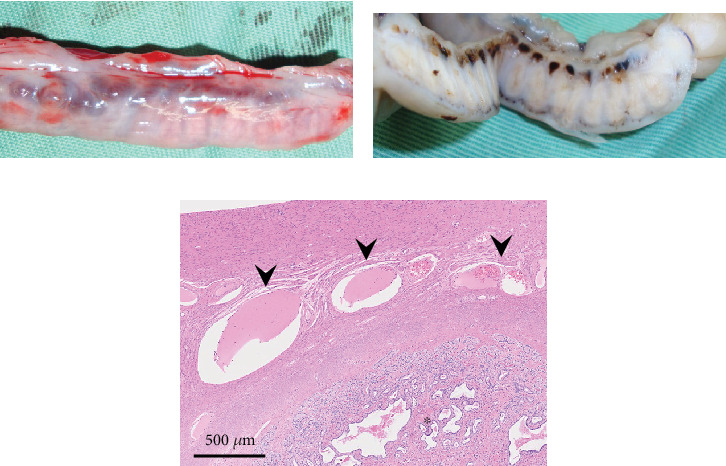
Gross and histological images of the uterine horn. (a) Severe dilation of blood vessels around the uterine horn is observed. (b) In the cross section of the formalin-fixed specimen, severe congestion and endometrial thickening are noted. (c) Histologically, severe congestion (arrowheads) and proliferation of the endometrium (asterisk) are observed (HE stain).

The resected cystic lesion contained a large blood clot and 108 mL of noncoagulated blood (hematocrit value 21%) (Figures 2(a), 2(b), and 2(c)). Cytology, bacterial culture, and hormonal examination of the fluid were not performed. The cystic structure was located between the right ovary and the cranial end of the right uterine horn (Figure 2(b)), and it was not continuous with the uterine lumen. The both uterine horns showed moderate enlargement with severe vasodilation and endometrial thickening (Figures 3(a) and 3(b)).

**Figure 4:**
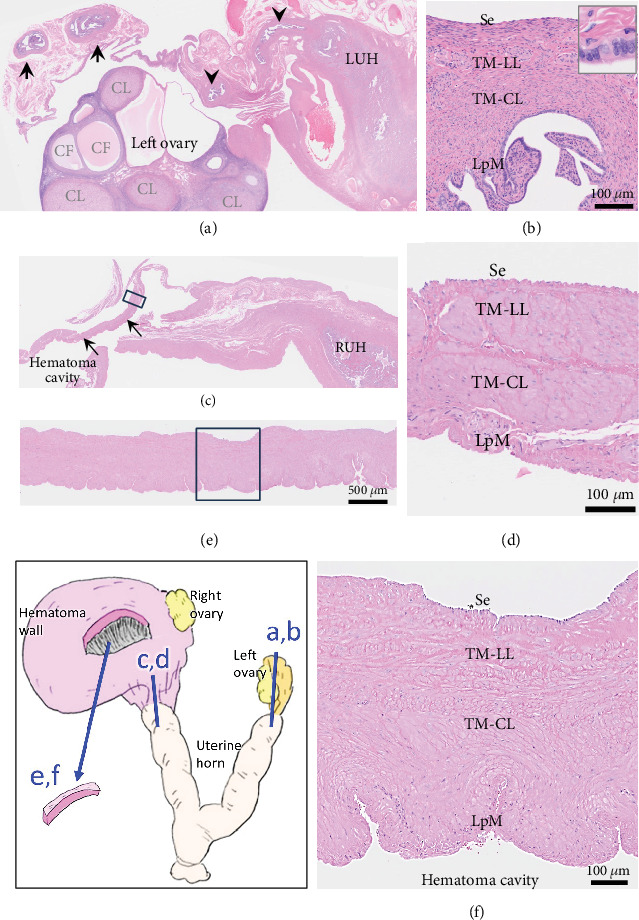
Histological images of the fallopian tube regions sampled from the area shown in the bottom-left illustration. (a) Longitudinal section of the left tube-uterine transition. The ampulla (arrows) and isthmus (arrowheads) of the fallopian tube with mucosal folds are identified cranial to the left uterine horn (LUH). The left ovary contains multiple corpora lutea (CL) and cystic follicles (CF). (b) Histological structure of the left fallopian tube isthmus, showing the serosa (Se), the outer longitudinal (TM-LL) and inner circular (TM-CL) layers of the tunica muscularis, and lamina propria of the mucosa (LpM) covered by cylindrical epithelium (box). (c) Longitudinal section of the right tube-uterine transition. No fallopian tube structures are identified cranial to the right uterine horn (RUH). A wall structure extending to the hematoma wall is observed (thin arrows). (d) Higher magnification of the boxed wall in (c). Layers include Se, TM-LL, TM-CL, and LpM, but epithelium and mucosal folds are absent. (e, f) The central hematoma wall (e) and higher magnification of the boxed area (f). The epithelium and mucosal folds are absent, but layers of Se, TM-LL, TM-CL, and LpM are present. The wall thickness is markedly greater than that of the left fallopian tube (b) or the wall near the RUH (d).

Histologically, the uterus was diagnosed with endometrial hyperplasia with severe congestion (Figure 3(c)). Both the right and left ovaries contained multiple cystic follicles and well-developed corpora lutea. Normal structures of the fallopian tube with mucosal folds were identified cranial to the left uterine horn (Figure 4(a)). The tube consisted of an outer longitudinal muscle layer, an inner circular muscle layer, and a lamina propria of the mucosa covered with columnar epithelium (Figure 4(b)). In contrast, in the right tube region, no fallopian tube with intact epithelium was identified (Figure 4(c)). Instead, the wall structure surrounding the hematoma cavity was observed to contain a lamina propria and two muscle layers (Figure 4(d)). A similar layered structure was also identified in the central portion of the hematoma wall, which was thicker than both the wall near the uterine horn and the fallopian tube wall (Figures 4(e) and 4(f)). These findings suggest that the hematoma wall is most likely a fallopian tube with eroded mucosal epithelium.

## 3. Discussion

There are limited veterinary reports on hematomas occurring in the ovaries, uterus, or their surrounding area. Ovarian hematomas in horses and cows have been reported to result from transvaginal follicular aspiration [2] or from the follicular bleeding after ovulation [1]. Uterine hematomas in dogs have been associated with cystic endometrial hyperplasia [3, 6], uterine torsion [3, 5], and rodenticide poisoning [4]. Broad ligament hematomas in cows and horses are caused by the ruptured uterine artery during delivery or assisted birth [8, 9]. Our feline case differed from these hematomas in both location and associated conditions, except for the presence of endometrial hyperplasia. Although blood sex hormone levels were not measured, the presence of multiple corpora lutea in the ovaries suggests that progesterone secretion may have induced endometrial hyperplasia [16].

Based on the gross location, the present case was consistent with hematosalpinx in humans, defined as bleeding into the fallopian tube [10, 12]. A huge cystic lesion, as in our case, has been reported [13, 14]. The fallopian tube in adult cats consists of the serosa, smooth muscle (tunica muscularis) composed of outer longitudinal and inner circular layers, and mucosa with a lamina propria covered by cylindrical epithelium [17]. The average thickness of the duct wall and muscularis was reported to be 212–325 and 200–280 *μ*m, respectively, while that of the epithelium was very thin (17 *μ*m) [17]. In the present case, no fallopian tube was observed in the right tube region, but the hematoma wall had tubal layer structures. In this region, the fallopian tube is the only structure with a similar layered architecture, making it reasonable to consider the hematoma wall as a fallopian tube with eroded epithelium. While fallopian tube epithelium can atrophy under progesterone influence [18], the preserved epithelium in the left tube suggests that the epithelial loss in the hematoma wall was likely due to significant dilation. Similar epithelial loss has been reported in huge hematosalpinx in humans [14]. The markedly thickened wall (> 700 *μ*m) at the most expanded area of the hematoma likely resulted from retraction of the stretched wall following hematoma removal (Figure 2(c)). The retained lamina propria and two muscle layers appear to have provided the strength to withstand the pressure from the massive hematoma.

Potential causes of hematosalpinx in humans include tubal pregnancy [12], ovarian or tubal torsion [14], tubal ligation [13], and malformations [11, 15] or tumors [10] in the uterus. Normally, the fallopian tube region receives blood from both the uterine and ovarian arteries. Therefore, even if blood flow from one source is disrupted by torsion, blood from the other source can still cause stasis and lead to hematoma formation [14]. Severe vasodilation observed in the uterine horn in our case suggested that blood flow disturbances might have been present. This finding in both horns indicates it was likely a contributing factor to hematoma formation, rather than a result of it. In humans, uterine malformations or tumors can cause menstrual blood to flow backward into the fallopian tubes, leading to hematosalpinx [10, 11, 15]. As no evidence of hemorrhage in the uterine cavity was observed in our case, retrograde blood flow from it is unlikely to be the cause.

Within the hematosalpinx, blood clots are generally observed [13, 14], but bloody fluid may also be present [11]. In the present case, ultrasound examination revealed a hypoechoic fluid component attached to a relatively hyperechoic solid region, which corresponded grossly to a bloody fluid and a blood clot, respectively. The erythroid regeneration observed at the time of presentation typically appears 2–5 days after bleeding [19], indicating that the hemorrhage had occurred earlier. The blood clot was likely due to older bleeding, and the addition of new bleeding (bloody fluid) may have caused the sudden collapse. As a rare case in humans, a large amount of bloody fluid leaking into the peritoneal cavity from a ruptured hematosalpinx has been reported [11]. Considering the risk of rupture and the severe condition of our case, we believe that emergency surgery was appropriate.

This case is the first report of hematosalpinx in a cat, characterized by a large hematoma in the fallopian tube. Since this condition can cause severe blood loss and sudden collapse, early ovariohysterectomy may be recommended. In this particular case, severe congestion of the uterus with endometrial hyperplasia could play a role in hematoma formation, but the exact cause is still unknown.

## Data Availability

The authors have nothing to report.
